# Preparation of high-concentration substitutional carbon-doped TiO_2_ film *via* a two-step method for high-performance photocatalysis

**DOI:** 10.1039/c8ra07082b

**Published:** 2018-10-30

**Authors:** Jun Wu, Xudong Jiang, Yupeng Zhang, Qiang Fu, Chunxu Pan

**Affiliations:** School of Physics and Technology, MOE Key Laboratory of Artificial Micro- and Nano-structures, Wuhan University Wuhan 430072 China cxpan@whu.edu.cn; Department of Conservation, Hubei Provincial Museum Wuhan 430077 China; College of Electronic Science and Technology, Shenzhen University Shenzhen 518000 China ypzhang@szu.edu.cn; Center for Electron Microscopy, Wuhan University Wuhan 430072 China

## Abstract

In this paper, we present a facile two-step method for preparing a high-concentration substitutional carbon-doped TiO_2_ (TiO_2−*x*_C_*x*_) film. First, the titanium substrate undergoes gas carburizing, followed by micro-arc oxidation (MAO) to form a carbon-doped TiO_2_ film on the surface. The process can be described as direct oxidation of titanium carbide (O→TiC_*x*_). The experimental results reveal that compared with traditional thermal annealing, this process could increase the carbon doping concentration to 6.07 at% and *x* to 0.24 in TiO_2−*x*_C_*x*_. The TiO_2−*x*_C_*x*_ film exhibits a significant red-shift in the band-gap transition, a narrow band gap of 2.77 eV, and excellent photocatalytic performance, more than two times higher than that of undoped TiO_2_ film. This method is simple, efficient, economical, environmentally friendly, and adapts to mass production. This experimental strategy can also be used in preparing other doped elements.

## Abstract 2

In this paper, we present a facile two-step method for preparing a high-concentration substitutional carbon-doped TiO_2_ (TiO_2−*x*_C_*x*_) film.

## Graphical abstract

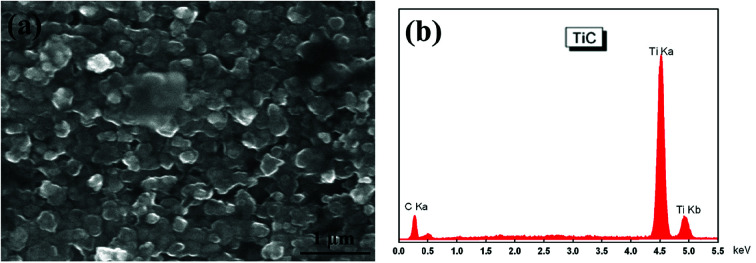

## 1. Introduction

In recent years, degradation of organic compounds by photocatalysis has become a very attractive research subject in the field of environmental pollution treatment. Different metal oxide semiconductors have been studied. Among various metal oxide photocatalysts, TiO_2_ is indicated to be an ideal photocatalyst with great application prospects because of its strong catalytic activity and high chemical stability.^1–3^ However, there are still some problems to be solved in practical applications, such as the low utilization of light and the recombination of photogenerated electron–hole pairs. In order to improve its photocatalytic performance, many methods have been proposed, such as metal ion doping,^4,5^ nonmetal ion doping,^6,7^ noble metal deposition,^8^ oxide semiconductor coupling,^9,10^ surface sensitization,^11^*etc.*

Theoretical and experimental studies demonstrate that nonmetal ion doping is one of the most common and effective methods to improve the photocatalytic performance of TiO_2_. Compared with other methods, non-metal doping is a kind of modification method with economic benefits. Irie *et al.*^12^ fabricated the carbon-doped TiO_2_ powders with red-shift adsorption spectra through direct thermal oxidation of TiC. It was found that carbon atoms were located at oxygen sites and formed the Ti–C bonds, but the carbon doping concentration in TiO_2_ was very low, only 0.32%. Park *et al.*^13^ obtained the TiO_2−*x*_C_*x*_ nanotube array by controlling the anodizing conditions, favoring the incorporation of carbon by heating with CO at temperatures up to 600 °C. The total photocurrent of the TiO_2−*x*_C_*x*_ nanotube array was more than 20 times higher than that of regular P-25. Wu *et al.*^14^ synthesized the carbon-doped TiO_2_ micro-/nanospheres and nanotubes *via* single-source chemical vapor deposition in an inert atmosphere. The estimated optical band gap was 2.78 eV for the carbon-doped TiO_2_ microspheres and 2.72 eV for the carbon-doped TiO_2_ nanotubes, both of which were much smaller than that of bulk anatase TiO_2_ (3.20 eV). Dong *et al.*^15^ prepared carbon-doped TiO_2_ with high visible light activity using a green synthetic approach with glucose as carbon doping source. It was found that the visible light absorbance of the thermally treated sample could be broadened due to the increased content of doped carbon. Wu *et al.*^16^ prepared carbon-doped TiO_2_ powders by a facile calcination-assisted solvothermal method. The experimental results showed that the carbon-doped TiO_2_ samples exhibited not only a very good ability to destroy NO gas, but also an excellent ability to degrade methyl orange solution under visible light, much superior to P25 and N–TiO_2_. Rasoulnezhad *et al.*^17^ synthesized carbon-doped TiO_2_ thin films on glass substrate by a combination of chemical vapor deposition and ultrasonic methods. It was found that carbon doping in the TiO_2_ structure greatly improved the optical properties for visible light absorption.

In general, the doping content of nonmetal-doped TiO_2_ films produced using the regular doping method is low, found mostly as interstitial doping, and has poor thermal stability. In our previous work, various TiO_2_ composite films, such as TiO_2_/YAG:Ce^3+^ and TiO_2_/Eu_2_O_3_ composites, have been prepared *in situ* by using micro-arc oxidation (MAO) technique on the titanium or titanium alloy substrate for high photocatalytic performance.^18,19^ In particular, we reported a new process which combined the technique of plasma nitriding and MAO to simply prepare a high-concentration substitutional N-doped TiO_2_ photocatalytic film.^20^ The principle is that the rapid oxidation process of MAO can restrict the nitrogen atoms' escape from the substrate and maximally keep the nitrogen atoms in the TiO_2_ film. Moreover, due to the direct oxidation reaction of TiN, the Ti–N bond is retained to the maximum extent, and more substitutional N-doped TiO_2_ is obtained.

In this paper, a facile two-step method is applied to prepare substitutional carbon-doped TiO_2_ film with high doping content. That is, as an important thermochemical treatment, gas carburizing is firstly used to form a carburized layer with rich TiC compound upon the titanium (Ti) surface, and then MAO treatment is used to directly oxidize the TiC compound into carbon-doped TiO_2_ film. Because of the fast, high-temperature, and *in situ* MAO treatment, the resulting carbon doping is of high content and in the form of substitutional doping in the TiO_2_ crystal lattice as the TiO_2−*x*_C_*x*_ film. Compared to plasma nitriding, gas carburizing has the advantages of simpler operation, higher carburizing concentration, large area, and more potential industrial application, among others.

## 2. Experimental

The two-step process for preparing the TiO_2−*x*_C_*x*_ film is as follows: (1) preparation of the TiC_*x*_ compound on the Ti substrate *via* gas carburizing in a self-made tubular furnace, *i.e.*, the Ti substrate was placed in a quartz tube reactor, heated to 600 °C, kept in 30 sccm C_2_H_2_ for 20 h, then kept in closed C_2_H_2_ for 10 h, and finally cooled to room temperature, while 200 sccm Ar gas was continuously introduced into the quartz tube. (2) Preparation of the TiO_2−*x*_C_*x*_ film *via* MAO, *i.e.*, the carburized Ti substrate was used as anode in 5 L electrolyte (Na_3_PO_4_·12H_2_O 10 g L^−1^), and 400 V constant pressure mode was adopted. As a comparison, a pure TiO_2_ film was also prepared *via* MAO under the same experimental conditions on the Ti substrate. The detailed experimental conditions are listed in Table 1.

**Table 1:** Experimental conditions of MAO

Samples	Anode	Electrical parameter					Electrolyte
		Voltage (V)		Frequency (kHz)	Duty cycle (%)	Time (min)	Na_3_PO_4_·12H_2_O (g L^−1^)
		Positive	Negative				
TiO_2_	Ti	400	0	1	20	3	10
TiO_2−*x*_C_*x*_	TiC_*x*_	400	0	1	20	3	10

The morphology and chemical compositions of the samples were characterized using a scanning electron microscope (Sirion SEM; FEI, Eindhoven, The Netherlands) with an energy-dispersive X-ray spectrometer (EDS). The phase analysis proceeded using an X-ray diffraction spectrometer (XRD) (D8 Advanced XRD; Bruker AXS, Karlsruhe, Germany) with Cu Kα source. The quantitative elemental compositions were analyzed by X-ray photoelectron spectroscopy (XPS) (VG Multilab 2000, Thermo Scientific, UK). UV-Vis diffuse reflectance spectra (DRS) of the samples were measured by using a diffuse reflectance accessory of the UV-Vis spectrophotometer (UV-2550; Shimadzu, Kyoto, Japan).

The photocatalytic performance was tested according to the following process: a 450 W high-pressure mercury lamp was used as light source, and 1 × 1 cm^2^ samples were placed in a quartz colorimetric dish containing 2 mL terephthalic acid (TA) solution (10 mM NaOH and 3 mM C_8_H_6_O_4_). Changes in the 2-hydroxy-terephthalic acid (TAOH) concentration were measured by UV-Vis spectrophotometer (UV-2550; Shimadzu) every 60 min.

## 3. Results and discussion

**Fig. 1:**
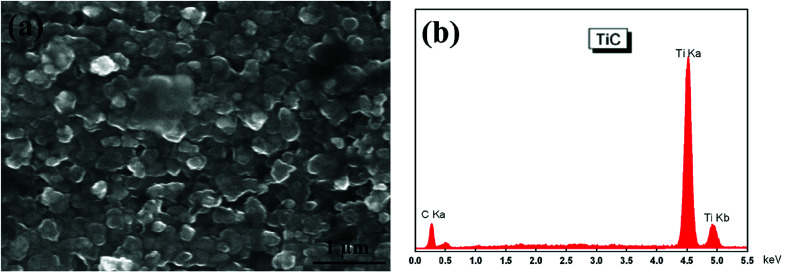
Surface characteristics of the carburized layer: (a) SEM morphology, (b) EDS profile.

**Fig. 2:**
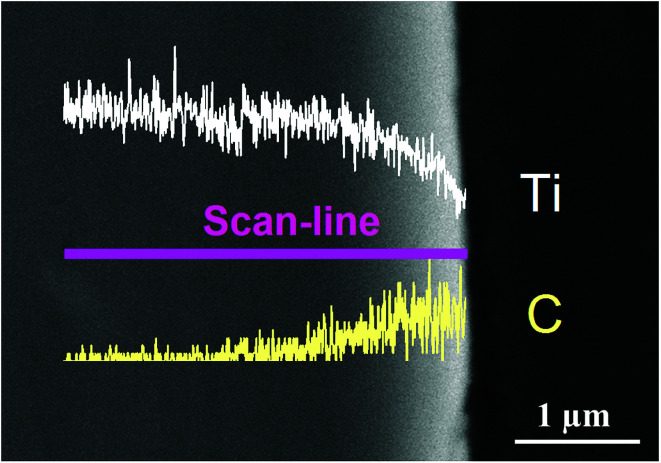
EDS line-scanning profiles of the carburized layer cross-section.

**Fig. 3:**
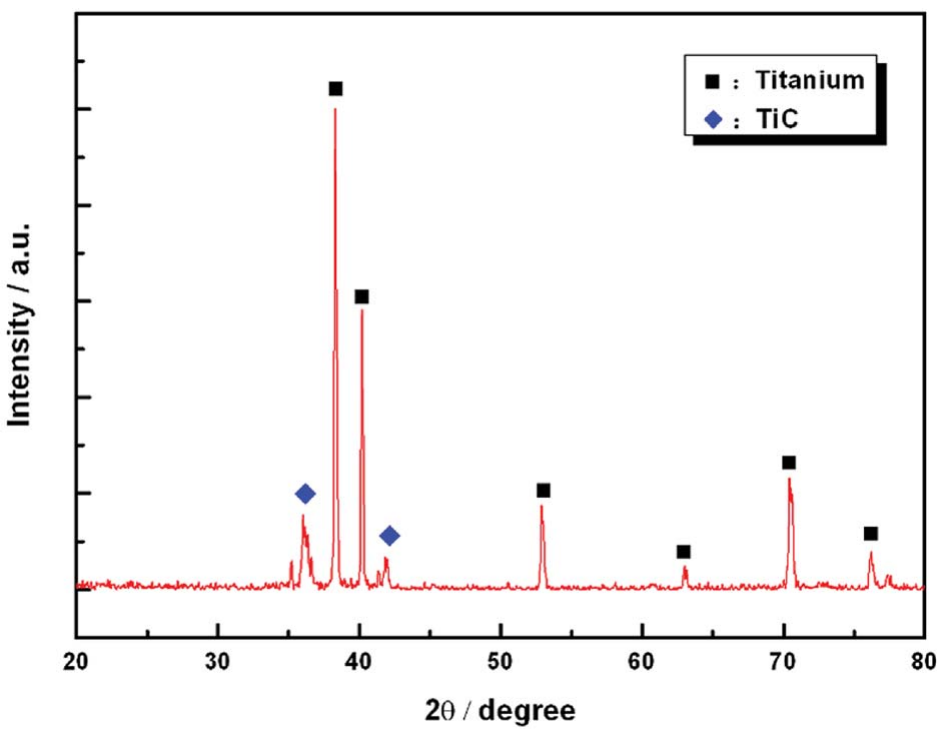
XRD pattern of the carburized layer.

Fig. 1 shows the SEM morphology and EDS profile of the gas carburized Ti substrate. It could be seen that the surface is loose, and there are many submicron grain sizes. EDS analysis revealed that besides Ti element, carbon (C) peaks also appeared obviously, which indicated that C atoms were infiltrated into the Ti substrate. From the cross-section of the gas carburized Ti substrate, as shown in Fig. 2, the carburized layer was about 1 μm in thickness, and the Ti and C content changed with a opposite variation from the outside surface into the interior substrate. Fig. 3 shows the XRD pattern of the gas carburized Ti substrate; evidently, TiC phase existed in the carburized layer.

**Fig. 4:**
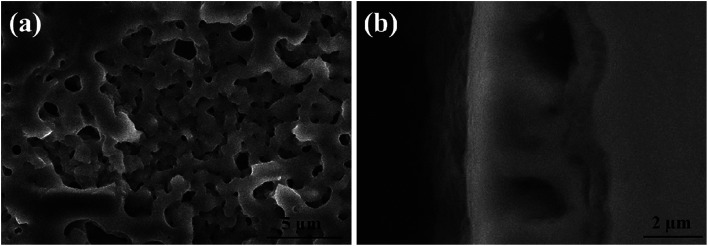
SEM morphologies of pure TiO_2_ film after MAO treatment: (a) surface, (b) cross-section.

**Fig. 5:**
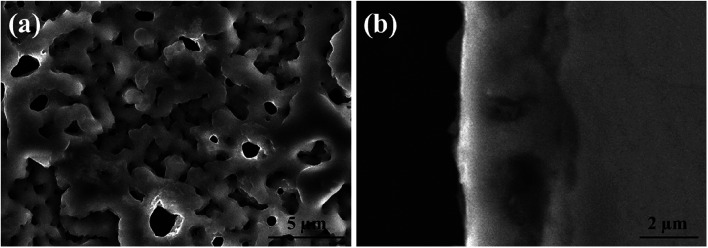
SEM morphologies of the TiO_2−*x*_C_*x*_ film after MAO treatment: (a) surface, (b) cross-section.

**Fig. 6:**
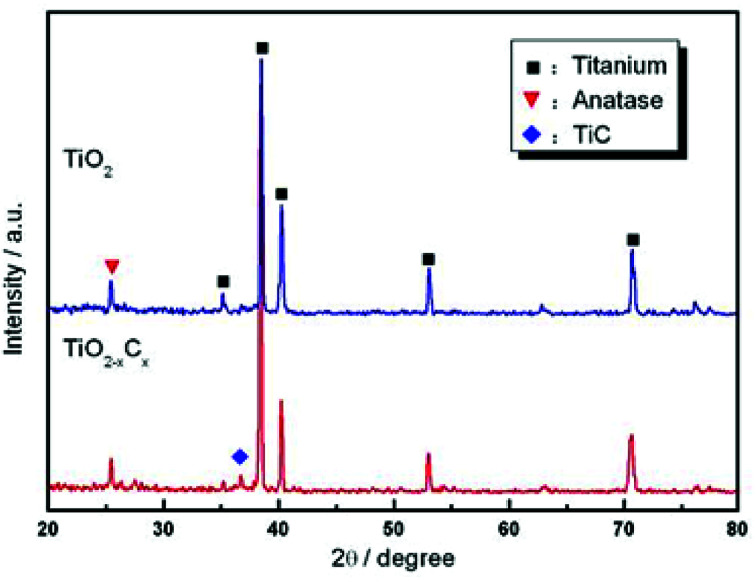
XRD patterns of pure TiO_2_ film and TiO_2−*x*_C_*x*_ film after MAO treatment.

Fig. 4 and 5 show the SEM morphologies of pure TiO_2_ film and the TiO_2−*x*_C_*x*_ film after MAO treatment, respectively. Both films showed no obvious difference; that is, all of the mesopores were separated well and distributed homogeneously over the film's surfaces with a diameter around 0.2–1 μm and thickness about 3–4 μm. It could be seen from Fig. 2 that the carburized layer was about 1 μm in thickness after gas carburizing. However, the coating thickness after MAO treatment was about 3–4 μm, which indicated a complete reaction in the carburized layer. Fig. 6 illustrates the XRD patterns of pure TiO_2_ film and the TiO_2−*x*_C_*x*_ film after MAO treatment, respectively. Both films were mainly composed of anatase phase, and no new phase was produced during doping. A small amount of TiC phase in the TiO_2−*x*_C_*x*_ film indicated that TiC was not completely oxidized to TiO_2−*x*_C_*x*_ during MAO treatment.

**Fig. 7:**
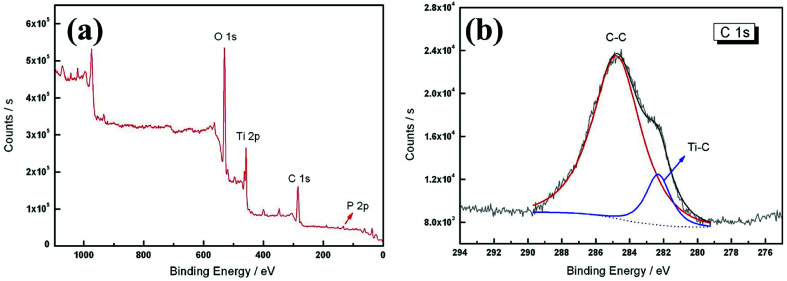
XPS spectra of the TiO_2−*x*_C_*x*_ film: (a) survey, (b) C 1s peaks.

XPS is an effective technique for verifying the concentration of elements on the surface of materials.^21^ In order to confirm the carbon atom site and content in the TiO_2−*x*_C_*x*_ film, XPS analysis was conducted, as shown in Fig. 7. Besides the element phosphorus (P) from the electrolyte, the existence of C element was clearly observed. In general, the doped C has two existing modes in the TiO_2_ lattice: (1) C replaces the position of oxygen (O) to form a O–Ti–C structure; (2) C is stabilized at an interstitial position.^22^ The multiplex high-resolution scans over the C 1s spectral region revealed two peaks at 284.8 and 282.3 eV, respectively. According to the commonly recognized C–C (285.3 eV) and Ti–C (281.9 eV) bonds,^23,24^ we believe that these two peaks belong to the C–C and Ti–C bonds, respectively. The C–C peak at 284.8 eV was the C peak for XPS calibration, not the peak of C in the sample, so its content was very high. Only the Ti–C peak at 282.3 eV was the peak of C in the TiO_2−*x*_C_*x*_ film. According to these XPS results, the O concentration was about 44.94 at%, compared with 44.10 at% of C. According to the area ratio of the C–C and Ti–C peaks, the concentration of Ti–C could be calculated as 6.07 at%. Then, *x* = 0.24 could be calculated from the generic formula of TiO_2−*x*_C_*x*_. Compared with the other methods, the present two-step process provided a possibility for both substitutional carbon-doping and high doping content. Table 2 lists the carbon doping data for various methods.

**Table 2:** Carbon content of the substitutional carbon-doped TiO_2_ produced by various methods

Catalyst	Method	Carbon content	Ref.
Carbon-doped TiO_2_ powders	Thermal oxidation	0.32%	12
TiO_2_–C1a/TiO_2_–C1b/TiO_2_–C2	Hydrolysis and calcination	2.98/0.42/0.03%	25
C–TiO_2_ film	Pulsed laser deposition	0.3%	26
C–TiO_2_/C–TiO_2_-200	Hydrothermal and annealing	0.19/0.28 at%	15
CT-BE/CE-BE-265	Calcination assisted solvothermal	0.6/0.42 at%	16
TiO_2_-1/TiO_2_-2/TiO_2_-3	CVD, hydrothermal, and annealing	2.3/2.8/3.9%	27
Carbon-doped TiO_2_ film	Gas carburizing and MAO	6.07 at%	Our work

**Fig. 8:**
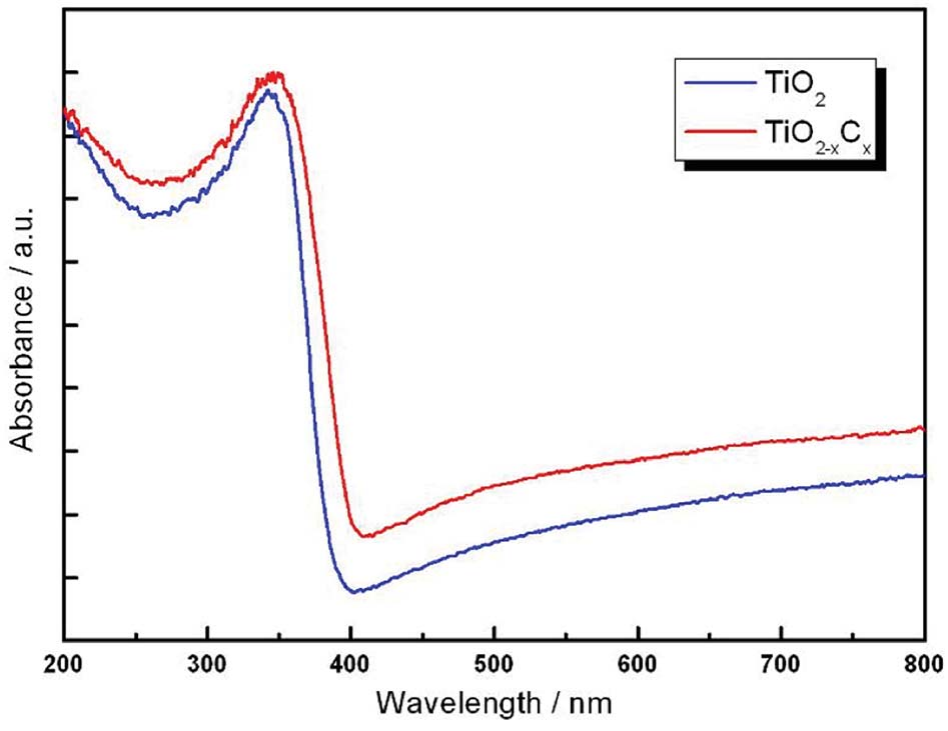
UV-Vis diffuse reflectance spectra (DRS) of pure TiO_2_ film and TiO_2−*x*_C_*x*_ film.

**Fig. 9:**
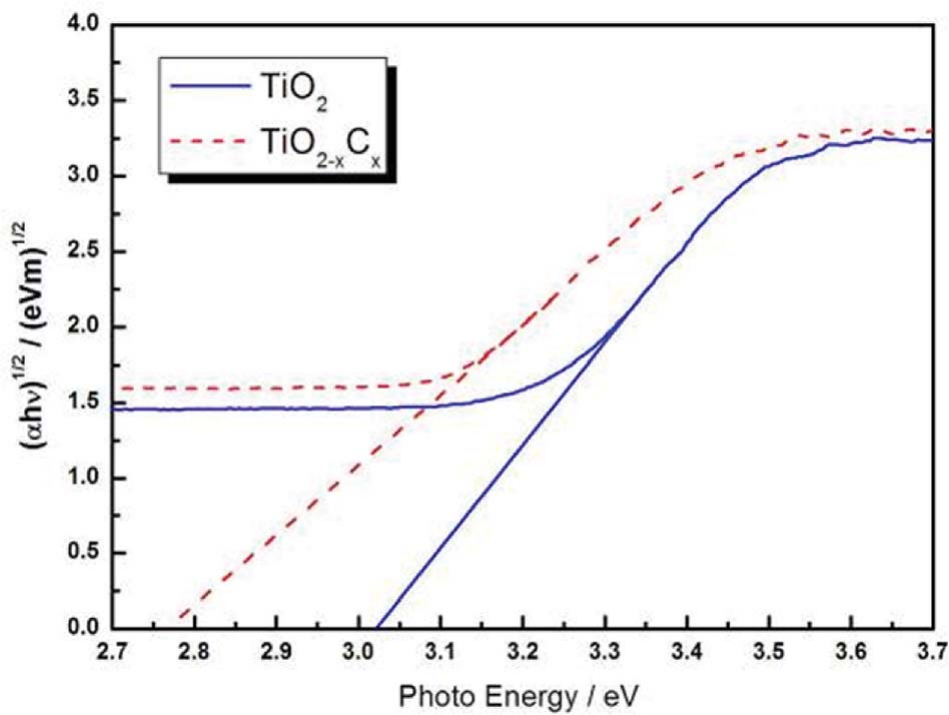
(*αhυ*)^1/2^ profiles as a function of photo energy for pure TiO_2_ film and TiO_2−*x*_C_*x*_ film.

Fig. 8 illustrates the DRS profiles of pure TiO_2_ and the TiO_2−*x*_C_*x*_ film. Obviously, the TiO_2−*x*_C_*x*_ film exhibited a red shift in the band gap transition and remarkably expanded wavelength response range to visible light; *i.e.*, the absorption edge of TiO_2−*x*_C_*x*_ film was shifted from the wavelength of 400 nm to 410 nm and exhibited a higher absorption. Using the Kubelka–Munk equations,^28^ the relationship between (*αhυ*)^1/2^ and the photo energy of the films can be determined. As shown in Fig. 9, the bandwidths of pure TiO_2_ film and TiO_2−*x*_C_*x*_ film were 3.02 eV and 2.77 eV, respectively, which demonstrates that the energy band-gap of TiO_2_ had been narrowed by C doping.

**Fig. 10:**
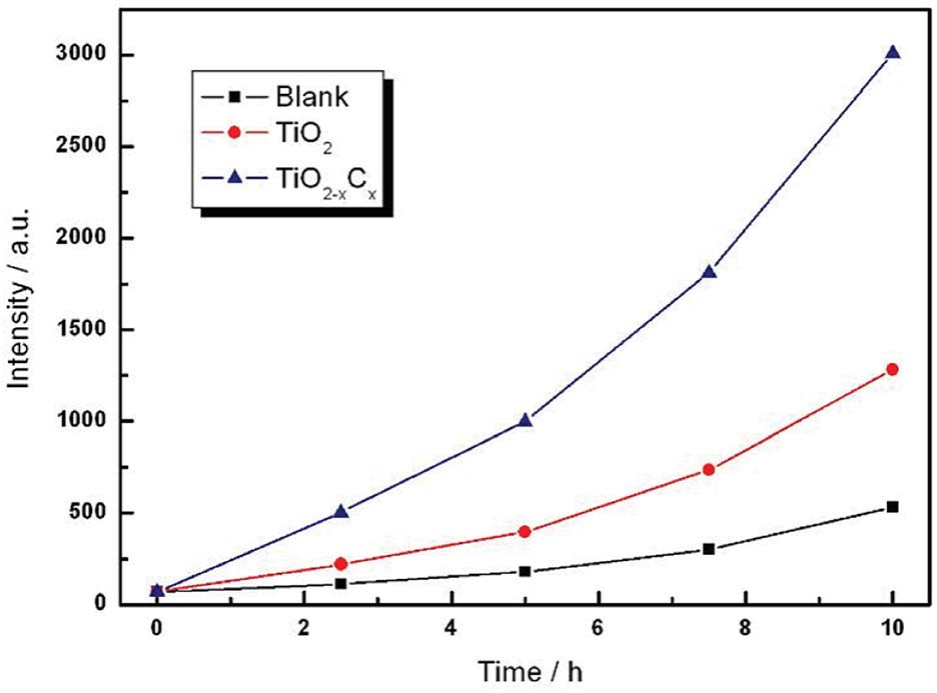
Time dependences of fluorescence intensity generated by pure TiO_2_ film and the TiO_2−*x*_C_*x*_ film on continuous illumination.

Further, we measured the content of ·OH to reflect the photocatalytic properties of the samples. Because TA can be combined with ·OH, the generated TAOH has a strong fluorescence emission peak at 426 nm, so it is used as a reagent for fluorescence detection. Fig. 10 shows the fluorescence intensity of TA solutions containing pure TiO_2_ film and TiO_2−*x*_C_*x*_ film at different illumination times. The experimental results indicated that the content of ·OH from the TiO_2−*x*_C_*x*_ film was about two times more than that of pure TiO_2_ film, and it exhibited a high photocatalytic performance.

In general, there are two kinds of strategies to prepare the C-doped TiO_2_, *i.e.*, incorporating carbon atoms into the TiO_2_ lattice (C→TiO_2_) and oxidizing the titanium carbide (O→TiC_*x*_). However, for preparation of the substitutional C-doped TiO_2_, the most used methods include direct thermal oxidation of TiC powders, pulsed laser deposition, chemical-vapor deposition, hydrothermal method, and annealing. Direct thermal oxidation of TiC powder is a simple one-step process, but the C doping concentration is low, and it is difficult to be recycled and can easily cause secondary pollution during photocatalytic degradation. Pulsed laser deposition and chemical-vapor deposition can also be a one-step process for producing the C-doped TiO_2_, but they usually require high energy under more stringent experimental conditions. Hydrothermal reaction and annealing are undoubtedly the most commonly used two-step method to prepare C-doped TiO_2_. However, some special reagents used in hydrothermal processes may pollute the environment, and the problem of low C concentration still exists.

For the present work, although the experiment is a two-step process, both gas carburizing and MAO are commonly used techniques in industry for surface modification of workpieces. Significantly, it can produce the substitutional C-doped TiO_2_ film with high concentration. Thus, it possesses the advantages of being simple, economical, and efficient, and the processes of gas carburizing and MAO causes no environmental problems. In addition, it adapts to mass production for the preparation of high-concentration substitutional C-doped TiO_2_ film, particularly with a large surface area.

## 4. Conclusions

A high-concentration substitutional carbon-doped TiO_2_ (TiO_2−*x*_C_*x*_) film is prepared *via* a novel two-step process, *i.e.*, combining gas carburizing thermochemical treatment and MAO techniques. The concentration of the Ti–C band reaches 6.07 at%, and the carbon doping amount is as high as *x* = 0.24. Therefore, the TiO_2−*x*_C_*x*_ film exhibits a significant red-shift in the band-gap transition, a narrow band gap of 2.77 eV, and excellent photocatalytic performance. This method is simple, efficient, economical, environmentally friendly, and adapts to mass production. According to this principle, doping with other materials, such as metal element doping, nonmetal element doping, metal and non-metal element co-doping, *etc.*, can be performed in the future.

## Conflicts of interest

There are no conflicts to declare.

## Supplementary Material
